# A high-cholesterol zebrafish diet promotes hypercholesterolemia and fasting-associated liver steatosis

**DOI:** 10.1016/j.jlr.2024.100637

**Published:** 2024-08-31

**Authors:** Yang Jin, Darby Kozan, Eric D. Young, Monica R. Hensley, Meng-Chieh Shen, Jia Wen, Tabea Moll, Jennifer L. Anderson, Hannah Kozan, John F. Rawls, Steven A. Farber

**Affiliations:** 1 Department of Embryology, Carnegie Institution for Science, Baltimore, MD, USA; 2 Department of Animal and Aquacultural Sciences, Norwegian University of Life Sciences, Aas, Norway; 3 Department of Biology, Johns Hopkins University, Baltimore, MD, USA; 4 Division of Gastrointestinal and Liver Pathology, Department of Pathology, Johns Hopkins Hospital, Baltimore, MD, USA; 5 Department of Molecular Genetics and Microbiology, Duke Microbiome Center, Duke University School of Medicine, Durham, NC, USA

**Keywords:** apolipoproteins, lipoprotein, adipocytes, liver, dietary cholesterol, hepatic steatosis, fatty acid synthase, fatty liver disease, dark liver, LipoGlo

## Abstract

Zebrafish are an ideal model organism to study lipid metabolism and to elucidate the molecular underpinnings of human lipid-associated disorders. Unlike murine models, to which various standardized high lipid diets such as a high-cholesterol diet (HCD) are available, there has yet to be a uniformly adopted zebrafish HCD protocol. In this study, we have developed an improved HCD protocol and thoroughly tested its impact on zebrafish lipid deposition and lipoprotein regulation in a dose- and time-dependent manner. The diet stability, reproducibility, and fish palatability were also validated. Fish fed HCD developed hypercholesterolemia as indicated by significantly elevated ApoB-containing lipoproteins (ApoB-LPs) and increased plasma levels of cholesterol and cholesterol esters. Feeding of the HCD to larvae for 8 days produced hepatic steatosis that became more stable and sever after 1 day of fasting and was associated with an opaque liver phenotype (dark under transmitted light). Unlike larvae, adult fish fed HCD for 14 days followed by a 3-day fast did not develop a stable fatty liver phenotype, though the fish had higher ApoB-LP levels in plasma and an upregulated lipogenesis gene *fasn* in adipose tissue. In conclusion, our HCD zebrafish protocol represents an effective and reliable approach for studying the temporal characteristics of the physiological and biochemical responses to high levels of dietary cholesterol and provides insights into the mechanisms that may underlie fatty liver disease.

## Background

Cholesterol is an essential sterol lipid and a structural component of cell membranes. Additionally, cholesterol serves as a precursor for steroid hormone and bile salt synthesis and impacts many cell signaling processes (1). Dietary cholesterol is absorbed by intestinal enterocytes, where it is esterified into cholesterol esters (CEs) for packaging into chylomicrons, an apolipoprotein B (ApoB) -containing lipoprotein (ApoB-LP), to be transported through the lymphatics and vasculature (2, 3). In circulation, ApoB-LPs vary by size and/or tissue of origin (eg, chylomicrons, VLDLs, IDLs, and LDLs). Each ApoB-LP contains one copy of ApoB per lipoprotein particle (4). Cholesterol is also transported by HDLs, which rely on apolipoprotein A1 as a structural component instead of ApoB. While the intestine is the first organ to process dietary cholesterol, the liver also plays a key role in regulating cholesterol homeostasis through a variety of processes: (1) uptake of cholesterol and CE from the circulation from lipoproteins like LDL, (2) conversion of cholesterol into bile salts for excretion, (3) packaging of cholesterol into VLDL for transport to peripheral tissues, and (4) synthesizing cholesterol de novo when dietary supplies are limited (5). Dysregulation of cholesterol homeostasis is linked to several lipid disorders including hypercholesterolemia, nonalcoholic fatty liver disease (NAFLD), coronary heart disease, type-2 diabetes, and obesity (6, 7, 8, 9, 10). The zebrafish has emerged as an ideal model for studying human lipid disorders such as hypercholesterolemia and NAFLD (11, 12). The digestive tract of zebrafish and mammals is highly similar and both use a similar lipoprotein transport system (13, 14). Furthermore, the small body size, fast growth, and optical transparency make the zebrafish larva optimal for live imaging and high-throughput in vivo drug discovery (15). Although traditional rodent models can exhibit elevated hypercholesterolemia in response to high dietary cholesterol, the cholesterol distribution between ApoB-LP and HDL differs between mouse and human (16). One explanation is that mice are naturally deficient in CE transport protein that transfers cholesterol from HDL to the highly atherogenic ApoB-LP (17, 18). On the other hand, the zebrafish expresses the *cetp* gene and have a more human-like lipoprotein profile than mice (19). A study by Stoletov *et.al.* (20) first showed that feeding HCD to zebrafish caused hypercholesterolemia, lipoprotein oxidation, and vascular lipid deposition. This work suggests that the zebrafish can be a powerful model for studying human lipoprotein regulation and associated lipid disorders. A recently developed zebrafish ApoB reporter line (LipoGlo) enables the study ApoB-LPs from individual larval zebrafish because it requires 1000-fold smaller amounts of plasma than previous assays (21). LipoGlo contains the Nanoluciferase sequence in the endogenous *apoBb.1* gene locus such that it produces an ApoB-Nanoluc fusion protein enabling the quantification of ApoB-LPs number, size, subcellular distribution, and tissue localization (21). Humans express ApoB isoforms that are intestinal-specific (APOB-48) and liver-specific (APOB-100), and both isoforms comprise the profile of ApoB-LPs in a volume of plasma. Zebrafish *apoBb.1* represents 95% of total *apoB* from both intestine and liver (22, 23). Thus, the LipoGlo zebrafish can be used to measure the quantity, distribution, and sizes of all classes of ApoB-LP (21). Another recently published zebrafish line that facilitates studies of lipid cell biology is the EGFP-*plin2* line which expresses a fluorescent fusion of EGFP to perilipin 2 (Plin2) from the endogenous gene locus (24). As PLINs are evolutionary conserved proteins that coat lipid droplets, this reporter line allows direct monitoring and quantification of the number and size of lipid droplets. Together the ApoB and Plin reporter lines enable us to track both intracellular lipids and plasma lipoproteins providing opportunities to evaluate the effect of HCD on zebrafish lipid physiology.

Different standard diets containing various combinations of cholesterol and other nutrients, such as carbohydrates and protein, have been extensively tested in murine models (25). Although custom-made HCD has been previously used for studying lipid metabolism in zebrafish (20, 26), there has yet to be a uniformly adopted protocol throughout the zebrafish field, and the effect of HCD in the zebrafish has not been thoroughly evaluated. In this study, we used an improved HCD protocol for zebrafish, replacing the ether solvent used to solubilize the cholesterol with less toxic ethanol in the preparation of the HCD. Our primary goal of this study was to address how dietary cholesterol alters lipoprotein regulation and lipid deposition in zebrafish. We found that the HCD increased ApoB-LP levels in a dose- and time-dependent manner. More interestingly, we have identified a fasting-dependent increase in hepatic steatosis in zebrafish larvae fed an HCD, suggesting a novel mechanism for cholesterol-induced lipid accumulation in the liver.

## Materials and Methods

### Ethics statement

All procedures using zebrafish were approved by the Carnegie Institution Department of Embryology Animal Care and Use Committee (Protocol #139).

### Zebrafish husbandry and maintenance

All zebrafish (*Danio rerio*) were raised and maintained in the zebrafish facility at the Carnegie Institution for Science, Baltimore, MD, United States. Zebrafish adults were maintained at 27°C on a 14:10 h light:dark cycle and fed once daily with ∼3.5% body weight GEMMA Micro 500 (Skretting). Embryos were collected by natural spawning and raised in a 28.5°C incubator with 14:10 h light:dark cycle before moving to the fish facility for initial feeding at 6-days post fertilization (dpf). Unless otherwise specified, the fish larvae were initially fed with GEMMA Micro 75 two times daily until 14 dpf. Juvenile (15–42 dpf) were fed with GEMMA Micro 150 twice per day and *Artemia* once daily. Fish older than 42dpf were fed with GEMMA Micro 300 once daily.

### Diet preparation

**Fig. 1:**
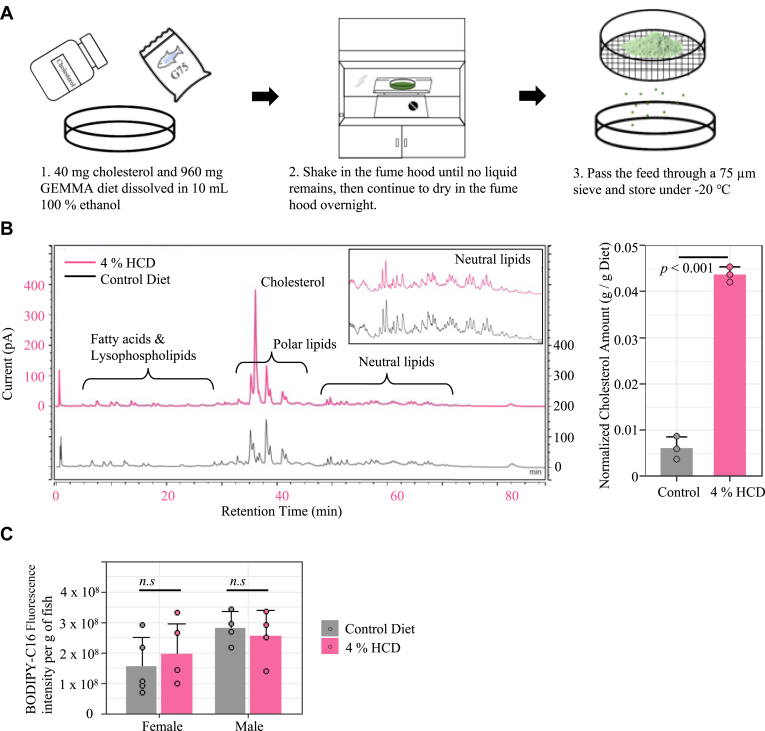
Diet-making protocol and quality control. A: Procedure for making the HCD. B: Quantification of lipids in diets using HPLC-CAD. The left figure shows representative chromatographs for extracted lipids from an HCD and the control diet. The bar graph on the right compares the cholesterol amount between HCD and the control diet (n = 3, mean ± SD, Welch *t* test). C: Comparison of food intake between fish fed HCD and control diet with 0.025% BODIPY-C16. Total fluorescence was measured from lipids extracted from the entire intestinal tract and liver of fish after 1 h of feeding (n > 4, mean ± SD, two-way ANOVA with Tukey HSD post hoc tests, n.s *P* > 0.05). HCD, high-cholesterol diet; HSD, honestly significant difference.

The 1%, 2%, 4%, and 8% cholesterol diets were made by dissolving 10 mg, 20 mg, 40 mg, and 80 mg of cholesterol (C8667, Sigma-Aldrich, St. Louis, MO), respectively, in 10 ml 100% ethanol (Fig. 1A). After the cholesterol was fully dissolved, 990 mg, 980 mg, 960 mg, and 920 mg of zebrafish diet (GEMMA, Skretting AS, Westbrook, Maine), respectively, was added. The mixture was shaken on a horizontal shaker in a fume hood until all visible liquid was evaporated, followed by leaving in the fume hood overnight ensuring that ethanol was fully evaporated. Dried HCD diet was pushed through a sieve (75 μm for G75, 150 μm for G150, and 500 μm for G500) using a pestle and stored at −20°C. The control diet was treated the same as the HCD, except no cholesterol was added. Fresh diets were made for each feeding trial.

### Feeding trials

All larval feeding trials were performed in a 3 L tank containing 60 zebrafish larvae. Eggs from same parents were incubated in petri dishes until 5 dpf before randomly distributed to 3 L tanks and started the feeding trial. Animals were fed either a control diet or an HCD from initial feeding at 6 dpf to 14 dpf. Two replicate tanks were used for each group. A time- and dose-response experiment was performed by feeding LipoGlo (*apoBb.1*^*NLuc/NLuc*^) (21) zebrafish either the control diet, 1%, 2%, 4%, or 8% HCD from initial feeding at 6 dpf to 14 dpf. Fish were sampled 2 h after the morning feeding at 6 dpf (after initial meal), 7 dpf, 9 dpf, and 14 dpf. All sampled fish were anesthetized with tricaine, and then placed under a stereo microscope to ensure the fish had food in their intestines. Four fish from each tank were immediately snap-frozen on dry ice, and then kept at −80°C until further LipoGlo-counting (21) was performed to measure total ApoB-LP concentration. Five fish from each tank were immediately imaged under a Nikon SMZ1500 microscope with an HR Plan Apo 1x WD 54 objective, Infinity 3 Lumenera camera and Infinity Analyze 6.5 software (www.lumenera.com). All fish were imaged under transmitted light with the same exposure and white balance settings. The experiment was repeated 3 times with 3 different clutches of animals obtained from natural crosses of LipoGlo adults.

A fasting experiment was done by feeding fish either control diet or 4% HCD from 6 dpf to 13 dpf. After their last meal, fish from two replicate tanks were merged and randomly split into two new tanks. One tank was fed the same HCD or control diet for another day, while the other tank fasted for 24 h or 48 h. The experiment was repeated three times using three different clutches of fish. One clutch of fish was fasted for 72 h. All fish from each day were imaged as described above.

Another fasting experiment was performed by initially feeding fish either control diet or 4% HCD from 6 dpf to 13 dpf. Six fish from each dietary group were transferred and fasted in a 12-well cell culture plate with 1 fish per well. The same individual fish was repeatedly imaged at 0 h, 15 h, 24 h and 48 h after last meal using an Axio zoom V16 microscope equipped with a Zeiss Plan NeoFluar Z 1x/0.25 FWD 56 mm objective, AxioCam MRm camera, and Zen 2.5 software (www.zeiss.com). The experiment was repeated 2 times with 2 different clutches of fish.

The adult feeding trial was conducted in 3 L tanks with no more than 30 fish per tank. One-year old fish were distributed equally into two 3 L tanks with equal gender per tank. The fish were fed either the control diet or HCD for 2 weeks. Postprandial fish were sampled 2 h after the last meal, while fasting fish further fasted for 3 days and then sampled at approximately the same time of day as compared to the sampling for postprandial fish. Sampled fish were anesthetized with tricaine fish tails were cut off to collect blood samples using EDTA coated Kunststoff-Kapillaren tubes (Sanguis Counting, Nümbrecht, Germany) and then transferred to 1.5 ml Eppendorf tubes. After centrifuging at 4°C for 2–5 min at 5,000 *g*, 1–3 μl of plasma were transferred into a new 1.5 ml Eppendorf tube and snap-frozen on dry ice. The middle intestine (27, 28) and liver were then dissected and snap-frozen on dry ice. A scalpel was used to take off the white skeletal tail muscle between the region of pelvic fins to the caudal part. Fish skin was carefully removed, and only upper half of the muscle was kept for further analysis. All samples were stored under −80°C before further analysis.

To compare the food intake between the 1-year-old fish fed the HCD and control diet, we used a fluorescent fatty acid (BODIPY-C16) as a dietary tracer and measured total fluorescent levels in the entire digestive tract (29). This was done by adding 0.5 mg (0.05%) of BODIPY-C16 to 1 g of the control diet or 4% HCD diet. Two tanks of fish, each with 5 females and 4 males, were fed either the control diet or 4% HCD containing 0.05% BODIPY-C16. The same protocol was used to prepare the diets except that BODIPY-C16 was added together with cholesterol during the process. A total amount of 0.315 g BODIPY-C16 diet was given to each tank, which was estimated as 3.5% of fish weight. After 2 h of feeding, the entire intestine with diet in the lumen was carefully dissected. Total lipids were extracted immediately for fluorescent measurement as previously described (29).

### LipoGlo assays

The generation of *apoBb.1*^*NLuc/Nluc*^ fish and analytical protocols (LipoGlo assays) were previously described (21). In brief, TALEN technology was used to fuse zebrafish *apoBb.1* gene with a complementary DNA (cDNA) sequence encoding NanoLuc® luciferase. These fish (*apoBb.1*^*Nluc/Nluc*^ or ^*Nluc/+*^ for the fusion protein) were used as described (21).

Sampled whole LipoGlo larvae or pieces of LipoGlo adult tissue were placed in individual wells of a 96-well plate and filled with 100 μl ApoB-LP stabilization buffer (21). Fish were then homogenized in a microplate-horn sonicator (Qsonica, Q700 sonicator with 431MPX microplate-horn assembly) filled with 17 mm of chilled, reverse osmosis water and processed at 100% power for a total of 30 s, delivered as 2 s pulses interspersed with 1 s pauses. Homogenates were stored on ice for immediate use, or frozen at −20°C and then thawed on ice for later use. Quantification of ApoB-LP levels (LipoGlo-Counting) was prepared by mixing 40 μl homogenate with an equal volume Nanoluc reaction buffer (PBS: Nano-Glo Buffer: Nano-Glo substrate furimazine, 30: 10: 0.2) in a black 96-well Perkin Elmer plate (6065400, PerkinElmer, Waltham, MA). Within 2 min of buffer addition, the plate was read under a SpectraMax M5 plate reader (Molecular Devices, San Jose, CA) with top-read chemiluminescent detection and a 500 ms integration time.

Three homogenates from each group were selected for LipoGlo-Electrophoresis, which was used to quantify the ApoB-LP size distribution as previously described (21). A 3% native polyacrylamide gel was prepared and cast overnight at 4°C using a 1 mm spacer plate and 10-well comb. The next day, the gel was put into a mini-protein electrophoresis rig filled with prechilled 1xTris-borate-EDTA (TBE) buffer at 4°C and pre-run at 50 V for 30 min. Afterward, 12.5 μl of sample solution (sample homogenate: 5x loading dye, 4: 1) was loaded per well. DiI-labeled human LDL (L3482, Thermo Fisher Scientific) solution (DiI-LDL: 5x loading dye, 4: 1) was used as a migration standard. The gel was run at 50 V for 30 min, followed by 125 V for 2 h. After the run, the gel was first equilibrated in an imaging solution (1 ml TBE + 2 μl Nano-Glo substrate) for 3 min, and then placed into an Odyssey Fc (LI-COR Biosciences) gel imaging system and imaged in the chemiluminescence channel for 2 min (NanoLuc detection) and then the 600 nm channel for 30 s (DiI LDL standard detection). The imaged gel was quantified using ImageJ (https://imagej.net/software/imagej/) based on the previously described method (21).

To visualize the whole-organism localization of ApoB-LP (LipoGlo-Microscopy), 14 dpf zebrafish larvae were anesthetized and fixed in 4% paraformaldehyde for 3 h at room temperature and rinsed 3 times for 15 min each in PBS-Tween (PBS containing 0.1% Tween-20 detergent). Fixed larvae were then mounted in 50 μl low melting point agarose (0.01 g/ml TBE) containing Nano-Glo substrate (1%). Nanoluc images were taken by Zeiss Plan NeoFluar Z 1x/0.25 FWD 56 mm objective, AxioCam MRm camera, and Zen 2.5 software (21).

### Oil Red O staining

The Oil Red O staining on whole zebrafish larvae was processed as described in (30). After fixing with 4% paraformaldehyde (PFA) in PBS overnight at 4°C, larvae were rinsed in 60% 2-propanol for 10 min and then put into 0.3% Oil Red O (Sigma-Aldrich, #00625) overnight at room temperature. Fish larvae were rinsed three times with 60% 2-propanol for 15 min before imaging.

The Oil Red O staining on larvae and adult fish sections was processed using modified method described in (31). Fish larvae or adult tissues were fixed in 4% PFA overnight at 4°C followed by infiltration with 30% sucrose overnight at 4°C. Fish larvae were embedded in Tissue-Tek OCT Compound (Sakura Japan Co., Ltd., Tokyo, Japan), frozen on dry ice and then 10–16 μm frozen sections were cut on a Leica CM3050 S Cryostat (Leica Biosystems, Deer Park, IL). Then, slides were dipped 2–3 times in 60% 2-propanol and stained with 0.3% Oil Red O for 20 min at room temperature. After staining, slides were dipped 2–3 times in 60% 2-propanol again and then rinsed with three dips in distilled water. After counterstaining with hematoxylin for 1 min, the slides were rinsed with tap water, coverslipped with an aqueous mounting medium and imaged immediately.

### Liver EGFP-Plin2 confocal imaging

We used the previously developed EGFP-Plin2 reporter line (24) to directly monitor and quantify the number and size of lipid droplets in zebrafish larvae. *Fus(EGFP-plin2)*/+ larvae were fed either 4% HCD or control diet from 6 dpf to 13 dpf, followed by fasting for 24 h. Confocal images of the liver were obtained from 4 fish fed HCD and 4 fish fed the control diet. Images were taken on a Leica SP5 II confocal microscope with a 63×1.4 HCX PL Apo oil immersion lens.

### Feeding trial on fabp6-GFP reporter line

To examine the effect of dietary cholesterol on zebrafish bile metabolism, we fed the fish 4% HCD and control diets and bile production was examined. A previously established *Tg(-1.7fabp6: GFP)* reporter line expresses GFP in the ileal epithelium driven by a 1.7-kb zebrafish *fabp6* promoter fragment that was used in the feeding trial (32). This reporter line can be used to monitor bile salt signaling in zebrafish because bile salts bind and activate farnesoid X receptor which is required to drive expression of *fabp6: GFP* (32). The feeding trial was performed in the fish facility in Duke University, Durham, NC, United States following protocols approved by the Duke University Medical Center Institutional Animal Care and Use Committee (protocol numbers A115-16-05 and A096-19-04). Since Cyp7a1 is the first and rate limiting step in bile salt synthesis pathway, we used transgenic mutant fish *cyp7a1*^*−/−*^*; Tg(-1.7fabp6: GFP)/+* and WT *cyp7a1*^*+/+*^*; Tg(-1.7fabp6: GFP)/+* (32) to monitor bile salt production. The fish were fed either HCD or control diet from initial feeding at 6 dpf to 13 dpf, followed by fasting 24 h. The fluorescence in the transgenic reporter line was quantified using a Leica M205 FA stereomicroscope with exposure time and magnification as described previously (32).

### HPLC

The quantification of dietary lipids or lipid classes was performed by using high performance liquid chromatography system with a Dionex Corona Veo charged aerosol detector (HPLC-CAD from Thermo Fisher Scientific, Waltham, MA). The HPLC-CAD and analytical methods were previously described (33). Lipids were extracted from diet homogenates using the Bligh-Dyer method (34), then dried and resuspended in HPLC-grade isopropanol. Lipid components of each sample were detected by the HPLC system with Accucore C18 column (150 × 3.0 mm, 2.6 μm particle size) and a CAD. A gradient mobile phase was used for separating different lipid classes: 0–5 min = 0.8 ml/min in 98% mobile phase A (methanol-water-acetic acid, 750:250:4) and 2% mobile phase B (acetonitrile-acetic acid, 1,000:4); 5–35 min = 0.8–1.0 ml/min, 98% - 30% A, 2%–65% B, and 0%–5% mobile phase C (2-propanol); 35–45 min = 1.0 ml/min, 30% - 0% A, 65%–95% B, and 5% C; 45–73 min = 1.0 ml/min, 95% - 60% B and 5%–40% C; and 73–80 min = 1.0 ml/min, 60% B, and 40% C.

To further identify peaks from different lipid classes on HPLC, TLC was first used to separate lipid classes from total lipid extracts of tissue homogenates (33). Total lipid extracts were dried and resuspended in 50 μl isopropanol, after which 20 μl was loaded in one row of a 20 cm × 20 cm channeled silica plate (LK5D, Whatman, Maidstone, United Kingdom) and another 20 μl was loaded on the adjacent channel. Lipid standards of triolein, cholesterol oleate, phosphatidylcholine (PC), and phosphatidylethanolamine (PE) (all from Sigma-Aldrich) were loaded on the adjacent columns. The plate was first run in the polar solvent (ethanol: triethylamine: ddH2O: CHCl3, 27: 25: 6.4: 25) to halfway up of the plate (10 cm), air-dried, and then run in nonpolar solvent (petroleum ether: ethyl ether: acetic acid 64: 8: 0.8) to near the top of the plate. The whole plate was then air-dried and cut in half. One half of the plate with one replicate of each sample and standards were sprayed with chromic-sulfuric acid (5% w/v potassium dichromate solution in 40% v/v aqueous sulfuric acid solution) and placed in oven (>180°C) to char the lipids. After cooling down, the lipid could be visualized and identified based on the position of the lipid standards. Based on the position of lipid bands on the charred plate, the lipid bands of triglyceride (TG), CE, PC, and PE from the uncharred plate were separately scraped out into 10 ml glass tubes for lipid extraction (supplemental Fig. S1A). One milliliter of chloroform: methanol (2:1) was added into the tube with silica, which was vortexed and centrifuged for 5 min at 2000 g. Afterward, 0.8 ml upper liquid was carefully transferred into a new tube, and the whole process was repeated 3 times. The collected 2.4 ml chloroform: methanol (2:1) was centrifuged for 5 min at 2000 g again to ensure all silica was sedimented at the bottom. Two milliliters of upper liquid was carefully collected, dried under N2, resuspended in 50 μl isopropanol, and then subject to HPLC-CAD as described above (supplemental Fig. S1B).

### RNA isolation, quantitative RT-PCR, and transcriptomic sequencing (RNA-seq)

Total RNA was extracted from the livers (n = 16, 4 fish per sex per dietary group) or adipose tissue (n = 8, 4 female fish per dietary group) of adult fish fed 4% HCD or control diet for 2 weeks followed by fasting for 3 days. RNA was extracted using Direct-zol RNA Microprep Kits (R2061, Zymo Research, Irvine), according to the manufacturer’s instructions. The RNA concentration and integrity were measured by a NanoDrop One (Thermo Fisher Scientific) and a Bioanalyzer (Agilent Technologies, Santa Clara). All extracted RNA had integrity values higher than 8, which indicates sufficient RNA quality for sequencing. The cDNA sequencing libraries were prepared using a TruSeq Stranded mRNA Library Prep Kit (Illumina, San Diego) according to the manufacturer’s instructions. Libraries were sequenced using 75 bp single-end mRNA sequencing (RNA-seq) on Illumina MiSeq (Illumina, San Diego, CA) at the Department of Embryology, Carnegie Institution for Science (Baltimore, MD). An average of 40 million reads was acquired from each sample. The reads were aligned to the zebrafish genome (GRCz11) using STAR (34). The resulting .bam files were subsequently used to generate raw gene counts using *featureCounts* (35). Ensembl Gene IDs were used to identify genes in this study. The raw fastq files are publicly available on Sequence Read Archive with accession number PRJNA998935.

For quantitative RT-PCR, the cDNA was synthesized using the iScript cDNA Synthesis Kit (Bio-Rad Laboratories, Inc, 1708891). All cDNA samples were then prepared using SsoAdvanced Universal SYBR Green Supermix (Bio-Rad Laboratories, Inc, 1725271). The primer pairs targeting zebrafish *fasn* transcripts (forward: 5′-CTGGCCATGGTCCTTAAAGATGG-3′, reverse: 5′-AGTTGGCGAAGCCGTAGTTG-3′) were used for gene expression analysis and zebrafish 18 S gene (forward: 5′ - GAACGCCACTTGTCCCTCTA - 3′, reverse: 5′- GTTGGTGGAGCGATTTGTCT - 3′) was used as the reference gene. The quantitative RT-PCR was performed in triplicate for each sample with the Bio-Rad CFX96 Real-Time System with 45 cycles: 95°C for 15 s, 59°C for 20 s, and 72°C for 20 s. All results were analyzed with the Bio-Rad CFX Manager 3.0 software (www.bio-rad.com), and relative gene expression was calculated using the ΔΔCT method (36).

### Statistics

Differential expression analysis was performed using R (v.4.1.2, www.r-project.org) package edgeR (37). Only genes with a minimum count level of at least 1 count per million in more than 50% of samples from each tissue were kept for further differential expression analysis. A generalized linear model approach described in the edgeR manual was used to find differentially expressed genes (DEGs) between HCD and control groups in each sex separately. DEGs were determined if a gene had a false discovery rate adjusted *P* value (*q* value) < 0.05 and absolute log2 fold change (|Log2FC|) > 1.

All statistical analyses were performed using R v.4.1.2. All datasets were first subjected to Shapiro-Wilk’s test for normality. One-way or two-way analysis of variance (ANOVA) was used for testing the main effect, and Tukey’s honestly significant difference was then used for post hoc testing. If the data were not normally distributed, Welch’s ANOVA from R package WRS2 was used for testing the main effect and the Games-Howell method for post hoc testing.

## Results

### Generation of a HCD

We developed an improved HCD paradigm for zebrafish, which can be used for studying the impact of dietary lipids on lipoprotein metabolism and overall digestive organ physiology (Fig. 1A). We used a popular commercially available diet (GEMMA, Skretting, Norway) as our basal diet, which contains 14% lipids and 59% protein (https://zebrafish.skrettingusa.com/). Since GEMMA is not an open-source formulation, we analyzed its lipid profile and found that predominate lipids were phospholipids, and the basal cholesterol level was about 0.6% of the diet (Fig. 1B). A 4% HCD was made by dissolving 0.04 g of cholesterol in 100% ethanol before adding 0.96 g of GEMMA. This mixture was shaken in a fume hood until no liquid remained and further dried overnight in the fume hood. The dried food was ground through sieves (50–100 μm for G75, 150 μm for G150, and 500 μm for G500) to make the final HCD product (Fig. 1A). By analyzing the lipid profiles of the diet, we found that the 4% HCD contained an average of 4.3% of cholesterol in the diet, compared to 0.6% cholesterol in the control diet (Fig. 1B). This indicated that the HCD-making process is highly effective and reproducible as there was little variation between different batches. All other phospholipid and neutral peaks were identical between the control diet and 4% HCD (Fig. 1B).

To further evaluate the effect of dietary cholesterol and ethanol treatment on fish palatability, we used fluorescent fatty acid BODIPY-C16 (0.025% of feed) as a dietary tracer to measure total fluorescent levels in the entire digestive tract after a single meal (29). Adult zebrafish were fed either the control diet or 4% HCD for 1 meal, and whole guts, including gut contents, were dissected after 2 h to analyze fluorescence in lipid extracts. Fish fed either diet had similar levels of gut lipid fluorescence, indicating that the 4% HCD was equivalently palatable as the standard feed (Fig. 1C).

### Feeding HCD increases total ApoB-LP abundance in zebrafish larvae

**Fig. 2:**
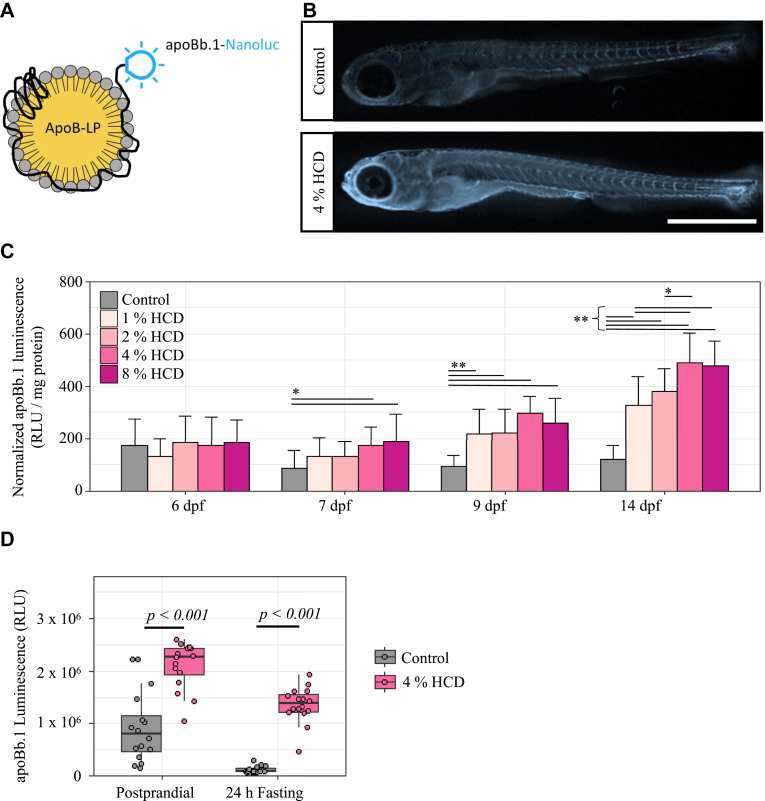
Effect of HCD on ApoB levels in zebrafish larvae. A: The LipoGlo reporter line, which contains an engineered *apoBb.1* gene fused with a nanoluciferase reporter was used for quantifying ApoB-LP levels in fish (21). B: LipoGlo-imaging was used to monitor ApoB-nluc chemiluminescence in whole zebrafish larvae (14 dpf) fed either the control diet or 4% HCD. Twenty-three fish were sampled from each dietary group and a representative image is shown. Scale = 1 mm. C: LipoGlo-counting quantified whole-body ApoB-LP levels in each diet from initial feeding at 6–14 dpf. Each bar represents mean ± SD from 24 fish (robust two-way ANOVA with Games-Howell tests). ∗*P* < 0.05, ∗∗*P* < 0.001 D: Comparison of ApoB-LP levels (LipoGlo-counting) in fish fed the HCD and control diet at postprandial or 24 h-fasted stage (n = 16 fish each group, robust two-way ANOVA with Games-Howell tests). ApoB, apolipoprotein B; ApoB-LP, ApoB-containing lipoprotein; dpf, days post fertilization; HCD, high-cholesterol diet.

To examine the effects of dietary cholesterol on ApoB-LP zebrafish physiology, we fed HCD to the LipoGlo fish through 14 dpf to monitor and quantify ApoB-LP using LipoGlo assays as previously described (21) (Fig. 2A). We found juvenile animals (14 dpf) fed a 4% HCD had increased overall ApoB-LP levels throughout the body as seen in the whole-mount LipoGlo images (Fig. 2B, 24 fish from each diet group). To determine if the HCD diets altered developmental growth rate, we measured fish standard length (SL) and height at anterior of anal fin (HAA) which can be used to evaluate overall developmental progress (38). Fish fed 4% HCD had similar SL (5.26 ± 0.46 mm) and HAA (0.34 ± 0.05 mm) as compared to fish fed the control diet (SL, 5.26 ± 0.50 mm; HAA, 0.34 ± 0.04 mm) (supplemental Fig. S2).

To further confirm that the increased ApoB-LP signals was indeed caused by the HCD, we fed *apoBb.1*^*Nluc/+*^ transgenic animals diets containing various concentrations of cholesterol (0%, 1%, 2%, 4%, and 8%) and quantified ApoB-LP (RLU per mg of protein) in whole zebrafish lysates from 6 to 14 dpf. After one day of feeding, at 7 dpf, the fish under the conditions of 4% and 8% HCD exhibited significantly (*P* < 0.05) higher ApoB-LP levels compared to fish fed the control diet (Fig. 2C). The ApoB-LP levels in fish fed 1% and 2% HCD appeared to increase later and were significantly (*P* < 0.05) higher than the control diet from 9 dpf onward. Furthermore, 14 dpf fish fed 4% and 8% HCD had significantly (*P* < 0.05) higher levels of ApoB-LP compared to fish fed 1% and 2% HCD (Fig. 2C). A 24 h-fast at 13 dpf significantly (*P* = 0.001, robust 2-way ANOVA) decreased ApoB-LP levels regardless of dietary cholesterol levels, and the levels of ApoB-LP remained significantly higher (*P* = 2 × 10^-5^, Games-Howell test) in fasted fish fed HCD compared to control diet (Fig. 2D).

A HCD protocol which used diethyl ether to deliver extra cholesterol to a standard fish diet was previously published (20). As diethyl ether is toxic to animals by inhalation or ingestion, we have improved the method by using more readily available and less hazardous ethanol to make the HCD. The efficiency of the two protocols was evaluated by comparing the dietary effect of HCD on ApoB-LP levels and indicated that ether- or ethanol-made HCD was able to increase the ApoB-LP levels in zebrafish larvae (14 dpf; supplemental Fig. S3). We also tested if vacuum treatment could improve the cholesterol-delivering efficiency of the HCD; however, no difference in ApoB-LP levels between the fish fed untreated and vacuum-treated HCD was observed (supplemental Fig. S3).

### Fasting after an HCD leads to liver steatosis

**Fig. 3:**
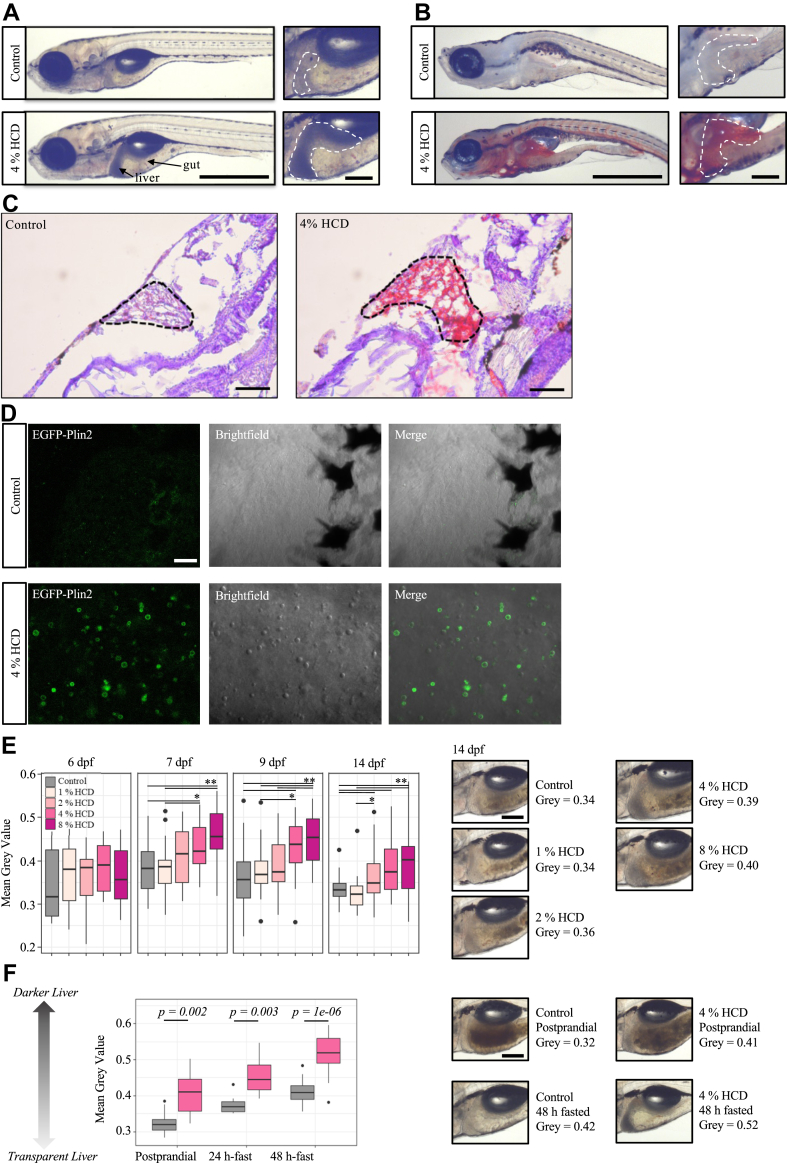
Effect of HCD on liver opacity of zebrafish larvae. A: Representative brightfield image of 14 dpf zebrafish larvae fed the control diet or 4% HCD from 6 dpf to 13 dpf followed by 24 h fasting (n > 20 fish per group). Scale = 1 mm. Scale for zoom image = 0.2 mm. B: Representative Oil-red-O staining image of 14 dpf zebrafish larvae fed the control diet or 4% HCD from 6 dpf to 13 dpf followed by 24 h fasting (n = 38 fish per group). Scale = 1 mm. Scale for zoom image = 0.2 mm. C: Representative Oil-red-O staining on slides of the zebrafish larvae fed the control diet or 4% HCD from 6 dpf to 13 dpf followed by 24 h fasting (n = 5 fish per group). Dotted line shows the liver region of the larvae. Scale = 0.1 mm. D: Confocal images of the liver of EGFP-Plin2 zebrafish, which express EGFP coding sequence fused to the N terminus of the endogenous *plin2* gene. Representative images were taken from the livers of 14 dpf fish fed 4% HCD or control diet (n = 4 fish per group). Scale = 10 μm. E: Measurement of liver opacity in postprandial fish (2 h after latest meal) fed control diet, 1%, 2%, 4%, or 8% HCD. Two hundred individuals were used in total. Liver opacity was calculated by measuring the mean gray value of the marked liver area (representative figures shown on the right), which were then normalized by the mean gray value of the muscle area next to the liver. A robust two-way ANOVA with Games-Howell tests was used for statistical test. Details of the method are illustrated in supplemental Fig. S4. Scale = 0.2 mm. F: Effect of fasting on liver opacity. Fish were fed either the control diet (gray) or 4% HCD (pink) from 6 dpf to 13 dpf, followed by either continued feeding or fasting for 24 h or 48 h. Liver opacity was calculated using the same method as D: A robust two-way ANOVA with Games-Howell tests was used for statistical test. Figures of the other two experiments are shown in supplemental Fig. S5. Scale = 0.2 mm. ∗*P* < 0.05, ∗∗*P* < 0.001. dpf, days post fertilization; HCD, high-cholesterol diet; Plin2, perilipin 2.

Larval zebrafish fed the 4% HCD from 6 dpf to 13 dpf then fasted for 24 h developed an opaque liver phenotype (dark under transmitted light; Fig. 3A, n > 20 fish from each diet group). After staining the whole fish with Oil Red O, 93% (37 out of 40 fish) exposed to the HCD showed staining in liver and 75% (30 out of 40 fish) showed staining in vasculature (Fig. 3B). The Oil Red O staining was quantified (ImageJ) to measure the mean gray value of the entire liver area, which was then normalized by the gray value of an adjacent area (supplemental Fig. S4). Feeding of 4% HCD had significantly (*P* < 0.05) higher Oil Red O staining than fish under the control diet (supplemental Fig. S5). Further H&E and Oil-red-O staining on sliced whole fish tissues have suggested that fish fed 4% HCD followed by fasting 24 h have higher intrahepatic lipid levels as compared to fish under the control diet (Fig. 3C). Since PLINs are evolutionally conserved proteins that coat lipid droplets, the *EGFP-plin2* reporter line allows us to visualize lipid droplets in live zebrafish at the tissue and subcellular level (24, 39). Therefore, we fed 4% HCD to *Fus(EGFP-plin2)/+* fish to visualize the subcellular location of the accumulated lipids (24). Indeed, more lipid droplets were observed in the liver of fish fed 4% HCD compared to the control diet (Fig. 3D).

To further characterize the liver steatosis observed in animals fed the HCD, we measured the liver opacity in postprandial fish fed various cholesterol concentrations (0%, 1%, 2%, 4%, and 8%). The opacity of the liver was quantified using ImageJ to measure the mean gray value of the entire liver area, which was then normalized by the gray value of an adjacent area (supplemental Fig. S4). Feeding of 4% and 8% HCD significantly (*P* < 0.05) increased the liver opacity from 7 dpf fish (Fig. 3E, n = 10 for each dietary group). No liver opacity differences were observed in fish fed the control diet, 1%, and 2% HCD, except that 14 dpf fish sometimes had a darker liver when fed 2% HCD than 1% HCD (*P* = 0.03, Fig. 3E). However, considerable variation was observed in the opacities of the livers between both individuals and different strains, for example, 30% (9 out of 30 fish) of the liver remained transparent in 14 dpf fish fed feeding 8% HCD. All fish were fed twice daily (08:00 in the morning and 13:00 in the afternoon). Since there is a 19 h interval between each afternoon meal and the next day morning meal, we hypothesize that fasting is another factor that influences liver opacity.

Therefore, an experiment was conducted for monitoring and quantifying liver steatosis associated with the degree of fasting. Fish were fed 4% HCD or control diet from 6 dpf to 13 dpf followed by fasting for 24 h or 48 h, while postprandial fish were fed the same diet up to 14 dpf. Although the liver opacity is significantly (*P* < 0.05) different between fish fed HCD and the control diet regardless of fasting, the level of differences was much larger in the 48 h-fasting group (*P* = 1 × 10^-6^, coefficient of variation = 0.1) than the postprandial group (*P* = 0.002, coefficient of variation = 0.14) (Fig. 3 F). By visualizing liver opacity under the microscope, we observed 100% dark liver (n = 19) in HCD-fed fish after 48 h fasting, while only 75% of the 24 h-fasted fish (9 out of 12 fish) and 64% of the postprandial HCD-fed fish (9 out of 14 fish) had dark livers. The fasting experiment was repeated 3 times with 3 fish stocks, and we observed the same results (supplemental Fig. S6).

Another fasting experiment was conducted to track liver steatosis in individual fish during fasting. Individual fish were imaged repeatedly at 0 h, 15 h, 24 h, and 48 h after fasting (n = 6 for each dietary group). A similar result was that the difference in liver opacity became larger after 48 h of fasting (*P* = 0.0007) than in postprandial fish (*P* = 0.009, supplemental Fig. S7). The experiment was repeated twice with two separate fish stocks. After 48 h of fasting, 92% (11 out of 12) of fish on the HCD developed dark liver, while the phenotype was not observed in control fish (supplemental Fig. S7). Both fasting experiments have suggested that fasting is highly associated with liver opacity.

### Feeding HCD followed by fasting causes hypercholesterolemia in adult zebrafish

To further investigate the effect of the HCD on the tissue and organ level, we have conducted an HCD feeding trial on one-year-old zebrafish adults. Zebrafish develop white adipose tissue which can store increasingly large amounts of neutral lipid as animals increase in size (40). Neutral lipid stored in adipose tissue in juvenile or adult zebrafish can be mobilized by fasting and eventually depleted as early as 7 days of fasting (41, 42, 43). Therefore, we speculated that adult fish under HCD needed more than 3 days of fasting to develop visible hepatic steatosis. We fed adult zebrafish 4% HCD or the control diet for two weeks, followed by a 3-days fast.

**Fig. 4:**
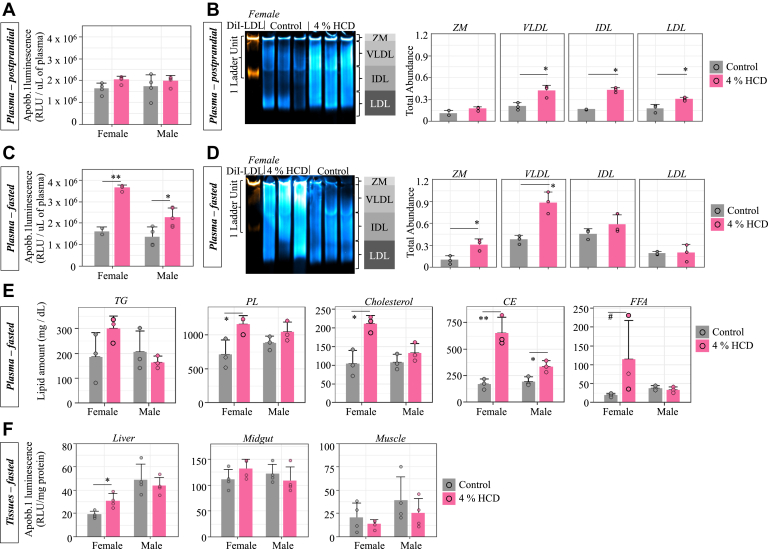
Effect of HCD on plasma ApoB-LP levels and plasma and liver lipids compositions of adult fish. A: LipoGlo-counting for measuring total ApoB-LP in plasma of postprandial fish fed the control diet or 4% HCD for 2 weeks (mean ± SD, two-way ANOVA with Tukey’s post hoc tests). B: The gel figure is LipoGlo-electrophoresis (a Native-PAGE assay to separate the ApoB-LP based on size) on 3 representative female plasmas from each diet (mean ± SD, student t-tests). Image is a composite of chemiluminescent (LipoGlo, blue) and fluorescent (DiI-LDL, orange) exposures. ApoB-LPs are separated into 4 classes based on mobility, including zero mobility (ZM), very-low-density lipoproteins (VLDLs), intermediate-density lipoproteins (IDLs), and low-density lipoprotein (LDL). The ZM fraction is a cluster of species above a certain size threshold, including chylomicrons and chylomicron remnants (21). Gel is a representative image from one of the three independent experiments performed. C: LipoGlo-Counting (mean ± SD, two-way ANOVA with Tukey’s post hoc tests). D: LipoGlo-Electrophoresis (mean ± SD, student t-tests) on plasma samples from fish fed the control diet and 4% HCD, followed by a 3-days fasting period. E: Quantification of major lipid classes in fasted zebrafish plasma using HPLC-CAD platform. A robust two-way ANOVA with Games-Howell tests was used for statistical test. F: LipoGlo-Counting for measuring total ApoB-LP in homogenized liver, gut, or muscle tissues of fasted fish after being fed the control diet or 4% HCD (mean ± SD, robust two-way ANOVA with Games-Howell tests). ∗*P* < 0.05, ∗∗*P* < 0.001, #*P* = 0.07. ApoB, apolipoprotein B; ApoB-LP, ApoB-containing lipoprotein; HCD, high-cholesterol diet.

No significant difference was observed for plasma ApoB-LP levels between control- and HCD-fed postprandial fish, though HCD female had slightly higher values on average (2 × 10^6^ vs. 1.6 × 10^6^ RLU/μl plasma, Fig. 4A). After fasting for 3 days, the HCD fish had significantly increased the ApoB-LP levels in plasma (*P* < 0.001 in females and *P* = 0.02 in males), while ApoB-LP levels in the fasted control fish remained the same as the postprandial controls (Fig. 4C). We subjected three female plasma samples from each dietary group to LipoGlo-Electrophoresis to characterize the ApoB-LP sizes (zero mobility, VLDL, IDL, and LDL) based on their migration distance (21). Gel images were transformed into ImageJ to calculate bin cutoffs for each ApoB-LP class based on the migration of the DiI standard. The absolute abundance of each ApoB-LP class was then measured by summarizing bin intensity values. The relative abundance was then calculated by transferring absolute abundance into percentage of total abundance for all ApoB-LP classes of each sample. Although postprandial fish fed the 4% HCD had significantly (*P* < 0.05) higher absolute abundance of VLDL, IDL, and LDL, the percentage of each ApoB-LP class remained the same between the HCD and control fish (Fig. 4B). The increase of ApoB-LP levels in female plasma reflects increased absolute abundance of VLDL and zero mobility classes, while the IDL and LDL levels were similar between fish fed HCD and control diet (Fig. 4D).

A previously established HPLC method was used to detect and quantify the composition of major lipids in plasma of zebrafish (44). Total lipids of zebrafish are separated into three major groups by HPLC, which are (1) free fatty acids (FFAs) and lysophospholipids, (2) phospholipids and cholesterol, and (3) triacylglycerol and CE (Fig. 1B). To further identify each individual peak, we used the TLC method (33) to separate PC, PE, TG, and CE. We then ran each lipid class on HPLC separately (supplemental Fig. S1). By summarizing all identified peaks of each lipid class, we have found that fasted HCD females had significantly higher (*P* < 0.05) plasma levels of phospholipids, cholesterol, and CE as compared to fasted control females, while only CE was significantly different between males fed HCD and the control diet (Fig. 4E). In addition, females fed HCD and then fasted likely had higher (*P* = 0.07) plasma FFA levels compared to females which fed the control diet before fasting (Fig. 4E). Feeding 4% HCD followed by fasting also increased ApoB-LP levels in female livers; however, no differences were observed in the intestine or muscle (Fig. 4F).

### Physiological and gene expression differences between liver of adult fish fed HCD

Unlike in larvae, feeding a 4% HCD followed by a fasting period resulted in unexpected variation in the steatotic liver phenotype. From H&E and Oil-red-O staining on fish liver slices, we observed some adults with dramatically increased number of hepatic lipid droplets in fasted as compared to postprandial fish; however, this finding was not consistent in all fish fed HCD (supplemental Fig. S8).

**Fig. 5:**
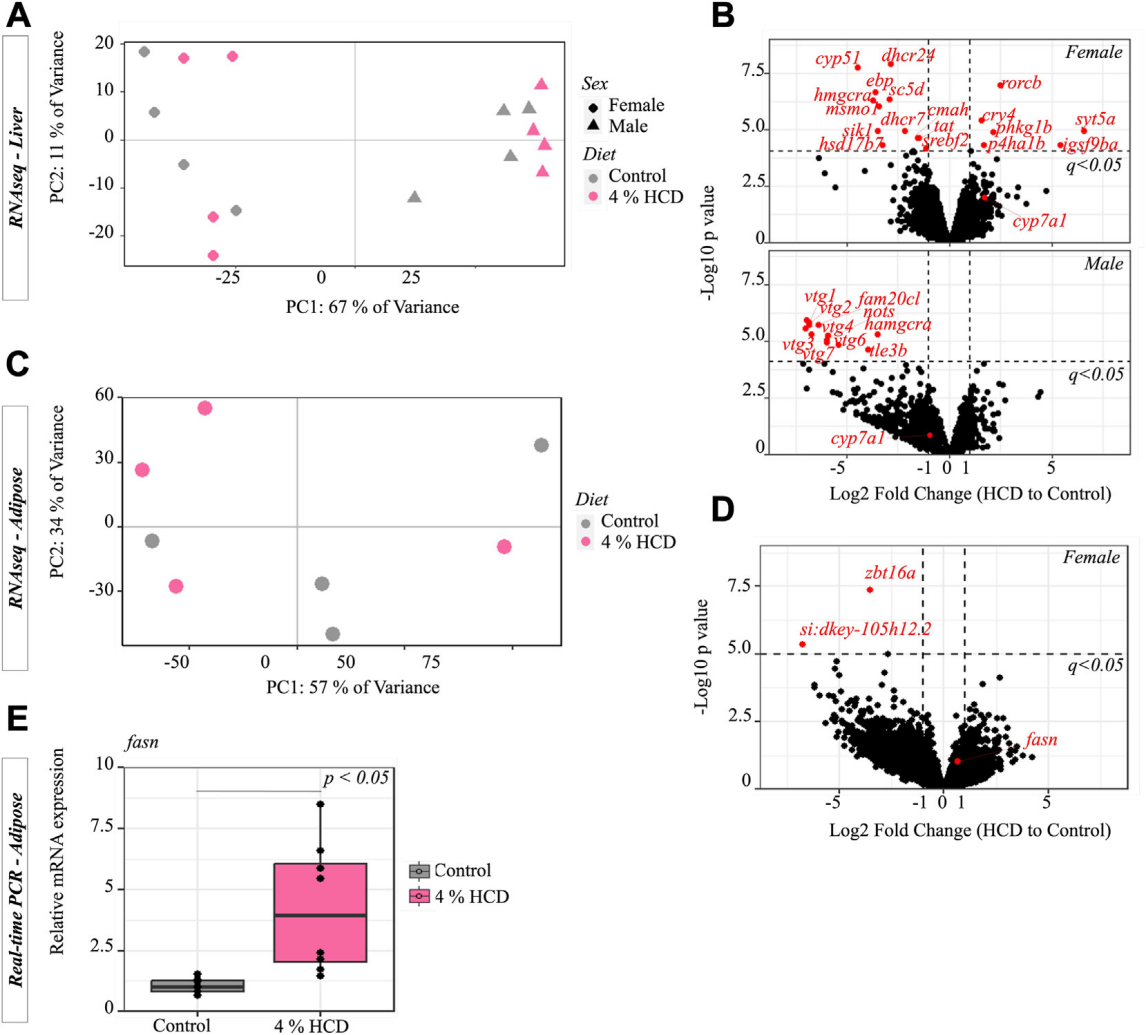
Gene expression analysis on adipose and liver of zebrafish fed 4% HCD and control diet followed by 3 days of fasting. A: Transcriptomic (RNAseq) analysis on the liver of fish fed 4% HCD compared to the control diet. The panel shows the score plot of PCA on log2 count per million (CPM) of the top 500 most variant genes across all 16 fish. B: DEGs (*q* < 0.05 and |Log2FC| > 1) between the livers of fish of each gender fed 4% HCD compared to the control diet. C: Transcriptomic analysis on the adipose tissue of fish fed 4% HCD compared to the control diet. The panel shows the score plot of PCA across all samples (4 individuals x 2 diets). D: DEGs between the livers of fish fed 4% HCD and the control diet. E: RT-PCR measurement of *fasn* gene expression (relative to control) in adipose tissue. Each diet group contains 8 individual fish samples (n = 8) collected from two independent experiments (Welch *t* test). DEGs, differentially expressed genes; HCD, high-cholesterol diet; PCA, principal component analysis.

To better understand the liver phenotype in adult animals fed the HCD, we performed RNAseq and found only a small number of differentially expressed genes (DEGs = 18 in females and 12 in males, *q* < 0.05 & |log2FC| > 1) (Fig. 5A, B). None of the DEGs were involved in TG synthesis or fatty acid oxidation pathways. Nine out of 12 downregulated DEGs in HCD females were involved in cholesterol biosynthesis pathways. Other downregulated DEGs included the *tyrosine aminotransferase* (*tat*) gene, which is involved in catalyzing the breakdown of L-tyrosine into p-hydroxyphenylpyruvate (45), and *cytidine monophospho-N-acetylneuraminic acid hydroxylase* (*cmah*), which hydroxylates N-acetylneuraminic acid (Neu5Ac), producing N-glycolylneuraminic acid (Neu5Gc) (46). Only 6 upregulated DEGs were identified in females fed the HCD compared to the control diet. These DEGs include 2 genes for circadian regulation (*RAR*-related orphan receptor C (*rorcb*) and cryptochrome circadian regulator 4 (*cry4*)). Other 3 DEGs were prolyl 4-hydroxylase subunit alpha 1 (*p4ha1b*), which encodes a key enzyme in collagen synthesis, phosphorylase B kinase gamma catalytic chain (*phkg1b*) which is involved in glycogenolysis, and the synaptotagmin 5a (*syt5a*) gene involved in the cellular response to calcium ions. All 12 DEGs were downregulated in HCD males compared to the control males (Fig. 5B). Half of these DEGs were *vitellogenin* (*vtg*) genes which are expressed at modest levels in male zebrafish (despite their well-described role in female lipid transport to the ovary) and may function as an additional lipid and protein transporters (19). Other interesting downregulated DEG includes 3-hydroxy-3-methylglutaryl coenzyme A reductase (*hmgcra*) which encodes the rate-controlling enzyme in cholesterol biosynthesis (47).

In vertebrates, fasting liberates FFA from adipocytes that are ultimately delivered to the liver, where they are essential for the synthesis of fuels needed to maintain organ function. Therefore, we measured expression of lipogenesis genes in female adipocytes that were fed either 4% HCD or the control diet following a 3-day fast. The quantitative PCR result showed that the key lipogenesis gene, fatty acid synthase (*fasn*) was significantly (*P* < 0.05, n = 8 fish per group) upregulated in fish fed HCD as compared to the control diet (Fig. 5E). To further investigate the gene regulatory network differences, we performed RNA-seq on abdominal visceral adipose tissue (40) of fish fed 4% HCD and the control diet, followed by fasting for 3 days (Fig. 5C, D). Only two differential expressed genes (DEG, *q* < 0.05 & |log2FC| > 1) were identified, which included the zinc finger and BTB domain-containing protein *16* (*zbt16a*) gene and an uncharacterized *si:dkey-105h12.2* gene. The expression of *fasn* gene was generally higher in fish fed 4% HCD than control diet, though the difference was not statistically significant (*q* > 0.05, Fig. 5D).

### Effect of HCD on bile salt production

**Fig. 6:**
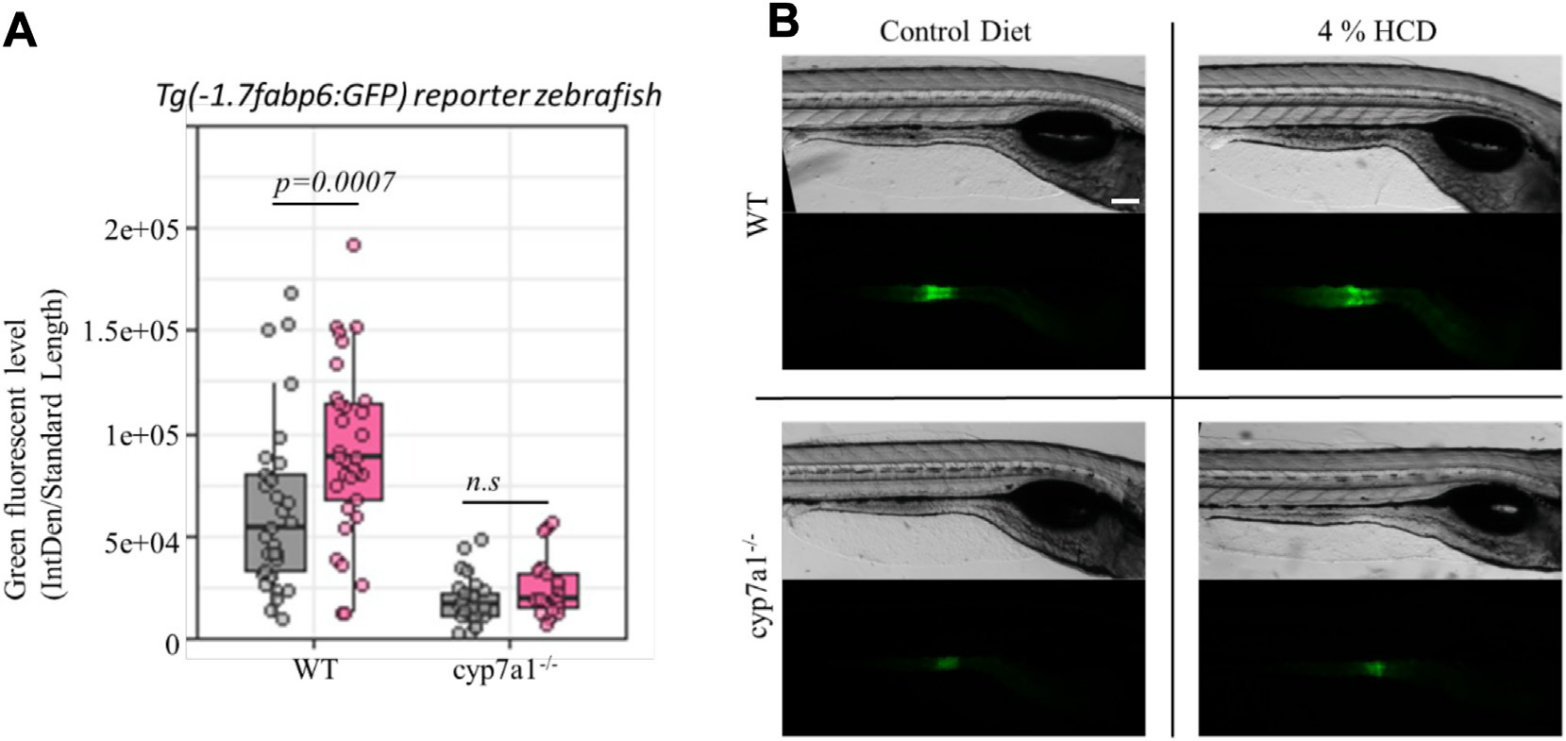
Effect of 4% HCD on bile acid signaling in zebrafish. A: Total green fluorescent protein fluorescence level in *Tg(-1.7fabp6:GFP)* reporter zebrafish with or without *cyp7a1* mutation fed either 4% or the control diet. As *fabp6* is an Fxr target, regulated by bile salts in zebrafish, this result suggests that our HCD could be used for modulating bile salt signaling in zebrafish. B: Representative brightfield and fluorescent images of WT and *cyp7a1* mutant larvae fed either control diet or 4 % HCD. Two-way robust ANOVA and Games–Howell test were used for all samples. Scale = 0.2 mm. Fxr, farnesoid X receptor; HCD, high-cholesterol diet.

Since bile salt production can be used to reduce systemic cholesterol levels in vertebrates (5), the effect of the HCD on bile production was examined. The previously established *fabp6:GFP* reporter line expresses GFP in the ileal epithelium driven by a 1.7-kb zebrafish *fabp6* promoter fragment (32). This reporter line can be used to monitor bile salt signaling in zebrafish because bile salts bind and activate farnesoid X receptor which in required to drive expression of *fabp6:GFP* (32). Indeed, we observed significantly (*P* < 0.05) higher GFP fluorescence in fish fed 4% HCD as compared to the control diet, which suggested that HCD alters bile signaling through modulation of bile acid synthesis (Fig. 6A, B). This HCD-induced fluorescent difference was attenuated in animals lacking the bile synthesis gene *cyp7a1* (Fig. 6A, B).

## Discussion

Excess dietary cholesterol has been associated with various lipid disorders, including hypercholesterolemia (48), NAFLD (8, 49, 50, 51), obesity, and diabetes (52, 53). This study describes a validated and reproducible HCD protocol for zebrafish that produced phenotypes consistent with hypercholesterolemia and NAFLD. Compared to the previously established HCD method, which mixes cholesterol and a basal fish diet in diethyl ether (11, 20, 54), our improved HCD method (1) has a more user-friendly detailed protocol where its stability, reproducibility, and fish palatability were validated, (2) improved the previously described protocol (20) by using a less hazardous solvent ethanol instead of diethyl ether, and (3) has been validated with one of the most widely used commercial zebrafish diets (GEMMA), of which we determined the lipid content (low TG and high phospholipids) (Fig. 1B). Zebrafish fed our HCD diet developed hypercholesterolemia, as indicated by a doubling of plasma cholesterol and ApoB-LP levels (Figs. 2 and 4). In humans, hypercholesterolemia is closely associated with atherosclerosis and high circulating LDL. Although we did not observe atherosclerotic plaques as others have previously described (20, 55), the increase of ApoB-LP, known to be atherogenic, justify the use of the zebrafish as a model for identifying factors that influence the development of human cardiovascular disease.

Another striking observation was that under the HCD, the zebrafish developed severe hepatic steatosis, as indicated by liver opacity (Fig. 3). Unlike previous studies which identified hepatic steatosis only using Oil Red O or Nile Red staining (26, 56), our HCD has introduced strong hepatic lipid droplet accumulation which scatters transmitted light (57) and produces the readily visible opaque liver phenotype under brightfield microscopy. Given that the zebrafish larvae are well-suited for high-throughput drug screening, this opaque liver phenotype provides a tractable opportunity to identify small molecules that attenuate liver steatosis.

The development of liver steatosis was strongly associated with fasting, suggesting that high dietary cholesterol and fasting interact synergistically to induce the metabolic and hepatic features that lead to steatosis (Fig. 3E, F). In mammals, fasting is characterized by the breakdown of TG stored in white adipose tissue into FFA, which then circulate in the plasma and are used by the liver and other organs to maintain energy homeostasis (41). If the FFA supply is overloaded or if the liver *β*-oxidation pathway is inhibited, liver FFA will be reesterified into TG and accumulate in cytoplasmic lipid droplets (58). This was supported by previous mice studies which identified liver-specific downregulation of *β*-oxidation gene *Cpt-1a*, VLDL synthesis gene *Mttp*, and lipogenesis gene *Fasn* of mice fed HCD (59, 60). However, our liver transcriptomic analysis did not identify DEGs in *de-novo* lipogenesis, *β*-oxidation, or VLDL synthesis pathways, in addition to the fact that we did not identify a consistent hepatic steatosis phenotype in adult fish fed HCD. One possible explanation for the contradictory results is that mice were exposed to an HCD for a much longer period (30 weeks) than adult zebrafish (2 weeks) which may alter liver lipid metabolism more severely. Nevertheless, we have found that adult fish fed 4% HCD exhibit higher expression of the key lipogenesis gene *fasn* in white adipose tissue and potentially higher FFA levels in plasma (*P* = 0.07). These data suggest that the HCD increases adipose tissue FFA synthesis that can then overflow into the plasma and then be taken into the liver during fasting.

In prior studies, knockdown of promyelocytic leukemia zinc finger protein (Plzf, encoded by *zbtb16* gene) was associated with increased adipogenesis and lipid accumulation in mouse adipose tissues (61). Our transcriptomic data showed that the *zbtb16a* gene was the most downregulated gene (Log2 fold change = −3.5, *q* < 0.001) in white adipose tissue in HCD-fed fish. Increased lipogenesis might provide HCD fish with a larger TG pool in adipocytes which supplies liver FFA during fasting. Mouse and primate studies have shown that increased dietary cholesterol was associated with adipocyte hypertrophy and increased free cholesterol (FC) content in visceral adipose tissue (52, 62). Adipocytes are known to be major vertebrate FC depot (49). Studies have also shown that the activation of lipolysis during fasting decreases the cellular level of TG in proportional to the cellular FC levels (62, 63). As intracellular FC is primarily stored in the lipid droplet surface layer (64), it is likely that hepatocyte lipid droplets need to maintain a FC:TG ratio and synthesize extra TG to balance the excess imported FC during fasting. Prolonged fasting also causes liver steatosis in animals under a normal diet, which is suggested to be caused by reduced liver VLDL production during fasting that limits TG efflux (65). However, the VLDL production is less likely the limiting factor for the zebrafish HCD-induced liver steatosis we observed since we found that plasma VLDL levels doubled in fish fed the HCD (Fig. 4A).

Previous studies have identified a zebrafish yolk opacity phenotype by mutating the key TG synthesis or transport genes (*mttp* (30) or *pla2g12b* (66)), which cause accumulation of TG that scatters light as it passes through the yolk syncytial layer. Our dark liver phenotype is also likely due to accumulated TG since the liver opacity is strongly associated with fasting and our transcriptomic data support the hypothesis that adipose derived FFA are being transported from plasma to liver to support TG synthesis. However, we still cannot rule out that accumulated cholesterol and CE could also cause dark liver in zebrafish (67). The analysis of liver transcriptome has identified decreased expression of key genes involved in *de-novo* cholesterol synthesis such as *hmgcra* and *dhcr24*. This was associated with higher levels of plasma cholesterol and CE in fish fed HCD, which suggested accumulated cholesterol and CE levels in liver inhibited the *de-novo* cholesterol synthesis pathway (5).

The early stages of NAFLD are characterized by simple hepatic steatosis, which can progress to a more aggressive stage called nonalcoholic steatohepatitis (NASH) that can then develop inflammation with or without fibrosis (68). As fibrosis develops, NASH can progress to cirrhosis, the end-stage of NAFLD. Although the molecular mechanism underlying the progress of NAFLD remains unclear, many studies have suggested that hepatic FC is critical for the development and progression to NAFLD (6, 8, 62). Our study has identified clear hepatic steatosis and higher levels of plasma FC, CE, and FFA in zebrafish under HCD (Fig. 4). These data suggest that the fish develop similar liver lipid phenotypes as those found in early stages of human NAFLD. Additionally, we have identified the upregulation of the *p4ha1* gene, which encodes a key enzyme in collagen synthesis (69). As liver fibrosis is characterized by excess deposition of collagen, the upregulation of *p4ha1* suggests that liver fibrosis may be induced in zebrafish under HCD. The zebrafish HCD model may have the potential to develop NASH by prolonging the fasting period or introducing additional dietary TG. Further, the inhibition of P4ha1 might decrease collagen production and work as a therapeutic for treating liver fibrosis (69, 70, 71).

In conclusion, our study has developed a well-validated HCD zebrafish larval model associated with increased ApoB-LP levels and fasting-associated liver steatosis, which makes it tractable for studying the underlying biology of hypercholesterolemia and NAFLD. Adult fish fed HCD developed hypercholesterolemia but not a consistent liver steatosis phenotype. The experimental strategies reported here further expand the zebrafish model system for studies of dyslipidemia and dietary-induced liver diseases.

## Data availability

Raw sequences are publicly available on Sequence Read Archive (SRA) under accession number PRJNA998935.

## Supplemental data

This article contains supplemental data.

## Conflict of interest

The authors declare that they have no conflicts of interest with the contents of this article.
