# Supplementary material for: A high-cholesterol zebrafish diet promotes hypercholesterolemia and fasting-associated liver steatosis
Source: J Lipid Res. 2024 Aug 31;65(10):100637. doi: 10.1016/j.jlr.2024.100637 (PMC11913794; doi:10.1016/j.jlr.2024.100637)
Supplement: Supplementary Figures S1–S8 [file mmc1.pdf]

A.

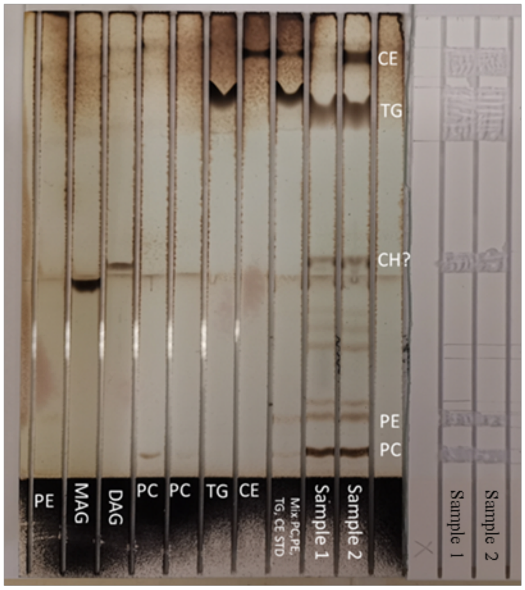

B.

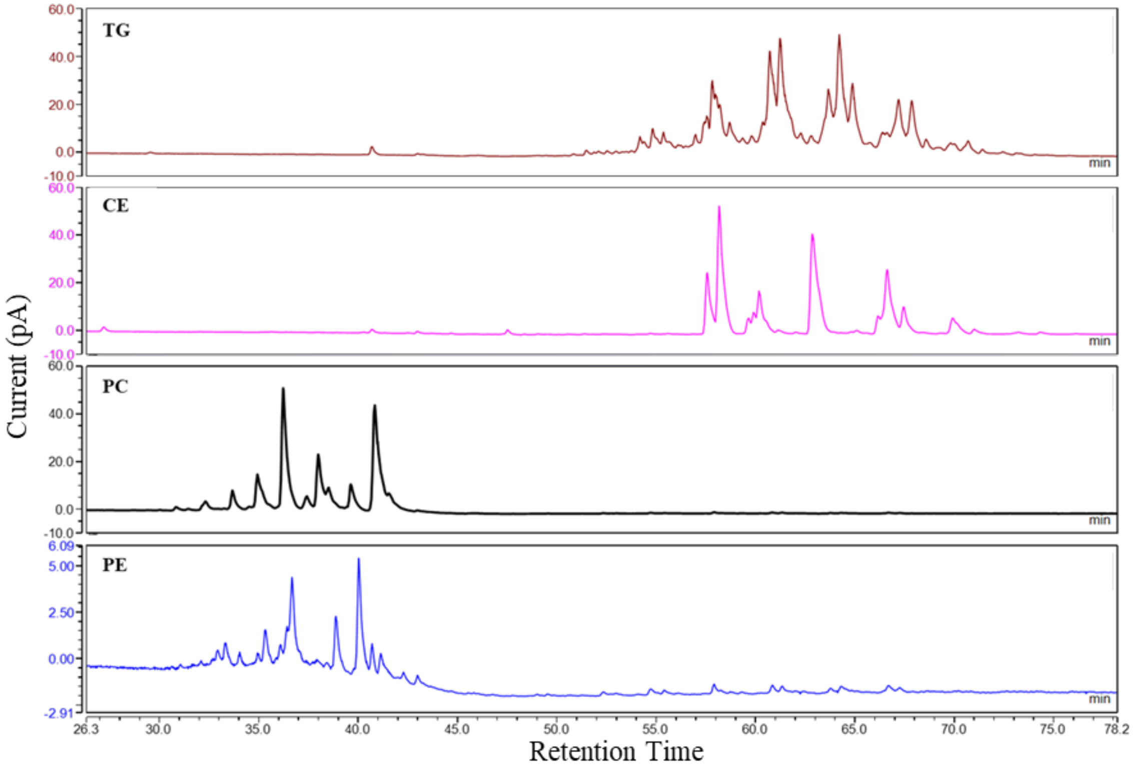

Supplementary Figure 1. A) A TLC plate which shows charred lipid standards and samples on the left and uncharred samples for HPLC analysis. **B)** Chromatogram figures for lipid classes separated from total lipids of zebrafish adult liver. Thin-layer chromatography (TLC) technology separated different lipid classes before running as separate samples on HPLC.

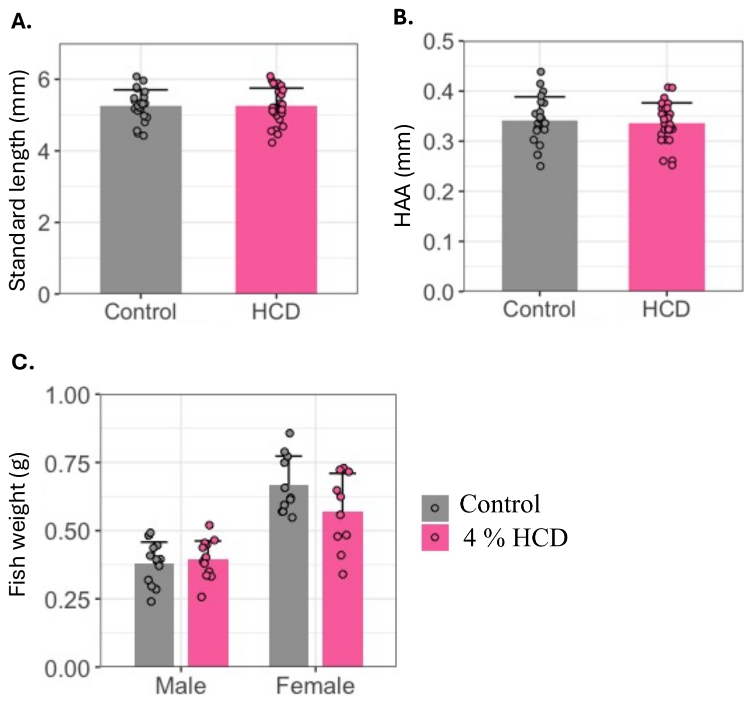

Supplementary Figure 2. Measurement of fish fed control diet and 4 % HCD. A) Standard length of larval fish fed HCD and control diet from 5dpf to 13dpf followed by fasting for 1 day**, B)** Anterior of anal fin (HAA) of larval fish fed HCD and control diet from 5dpf to 13dpf followed by fasting for 1 day, **C)** Weight of adult fish fed HCD and control diet for 2 weeks followed by fasting for 3 days**.** Over twenty fish were used in each dietary group (n > 20). Student t-test was used for all samples and no significant ($p > 0.05$) was found between fish fed control diet and 4 % HCD.

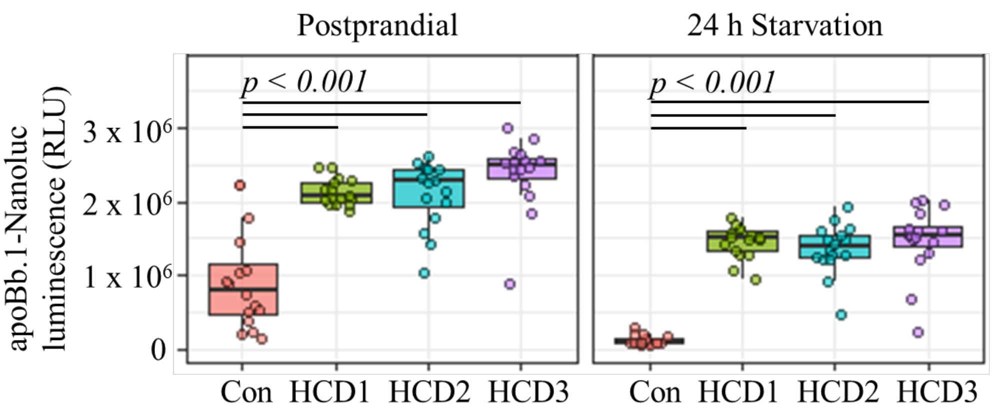

Supplementary Figure 3. Effect of HCD made by 3 different methods on total ApoB-LP levels (LipoGlo-counting) of 14 dpf fish. Control diet: standard GEMMA 75 diet treated with 100 % ethanol. HCD1: our standard HCD protocol (Figure 1 A), except that the mixture was vacuum treated before drying. HCD2: our standard HCD protocol (Figure 1A). HCD3: previously published HCD protocol which used ether to deliver cholesterol to GEMMA diet (20). Fifteen fish were used in each dietary group (n = 15). Two-way robust ANOVA and Games–Howell test was used for all samples.

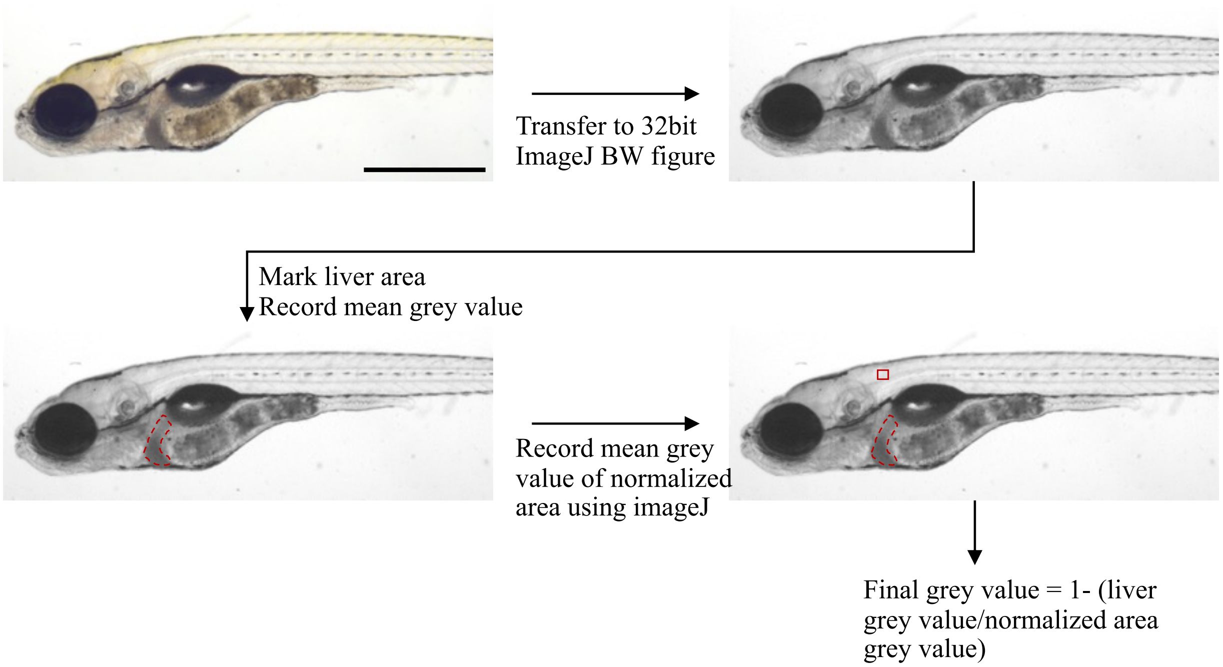

Supplementary Figure 4. Method for measuring opacity of the liver. Images were first transferred to 32-bit ImageJ black-and-white figures. Liver areas were marked, and mean grey values were measured using ImageJ. A 50x50 square was drawn in the fixed area in the muscle tissue near the liver for normalization. All images from the same day were taken under the same light intensity. Scale = 1mm.

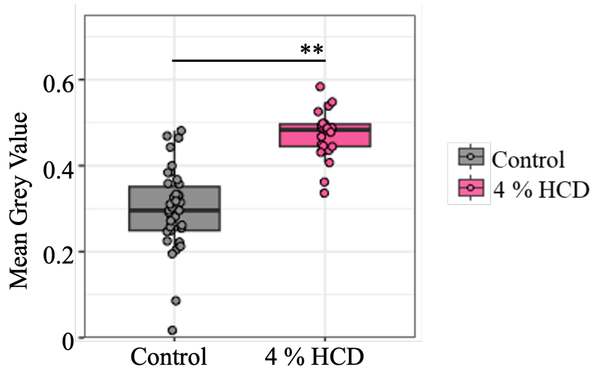

Supplementary Figure 5. Quantification of Oil Red O on fish fed 4% HCD and control diets.

Fish were fed either the control diet or 4 % HCD from 6 dpf to 13 dpf, followed by fasting for 24h. Figures were converted into black and white in Fiji and mean grey values from liver area were measured.

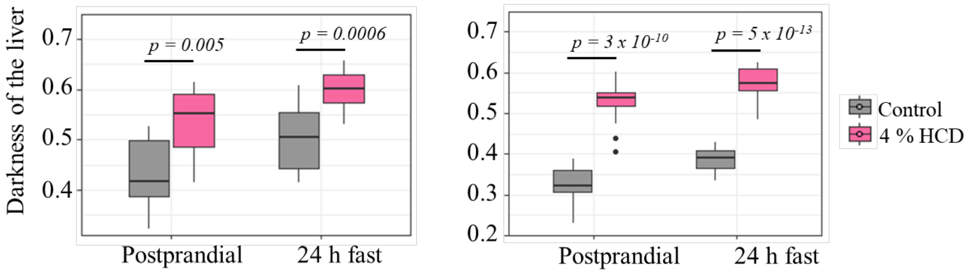

Supplementary Figure 6. **Effect of fasting on liver opacity between fish stocks.** Fish were fed either the control diet or 4 % HCD from 6 dpf to 13 dpf, followed by either continued feeding or fasting for 24h. Two-way robust ANOVA and Games–Howell test were used.

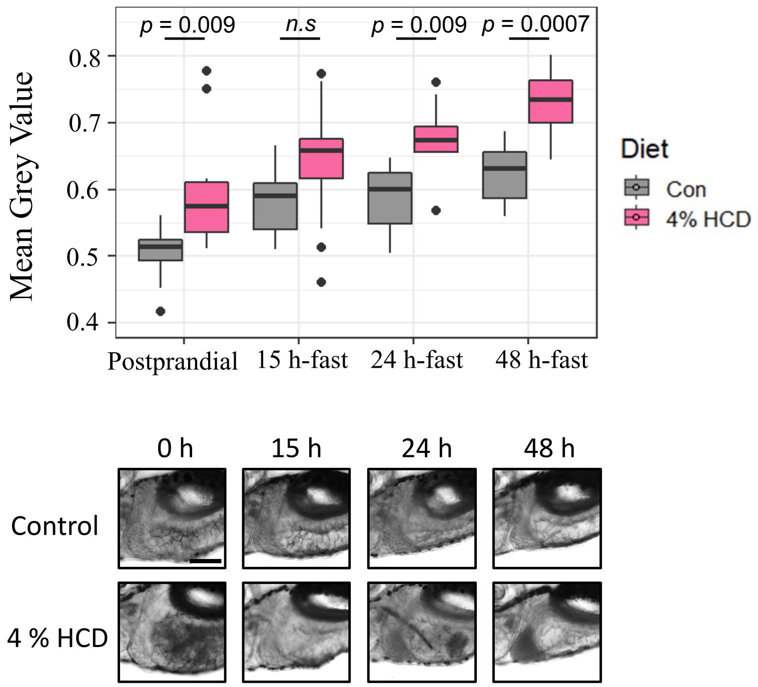

Supplementary Figure 7. Effect of fasting period on liver opacity. Fish were fed either the control diet or 4 % HCD from 6 dpf to 13 dpf, followed by fasting. Individual fish were tracked and imaged at 0 h, 15 h, 24 h, and 48 h after fasting (n = 6 per each diet group). The top panel is merged data from 2 independent experiments. Two-way robust ANOVA and Games–Howell test was used for all samples. The bottom panel is representative of individual images following fasting. Scale = 0.2 mm.

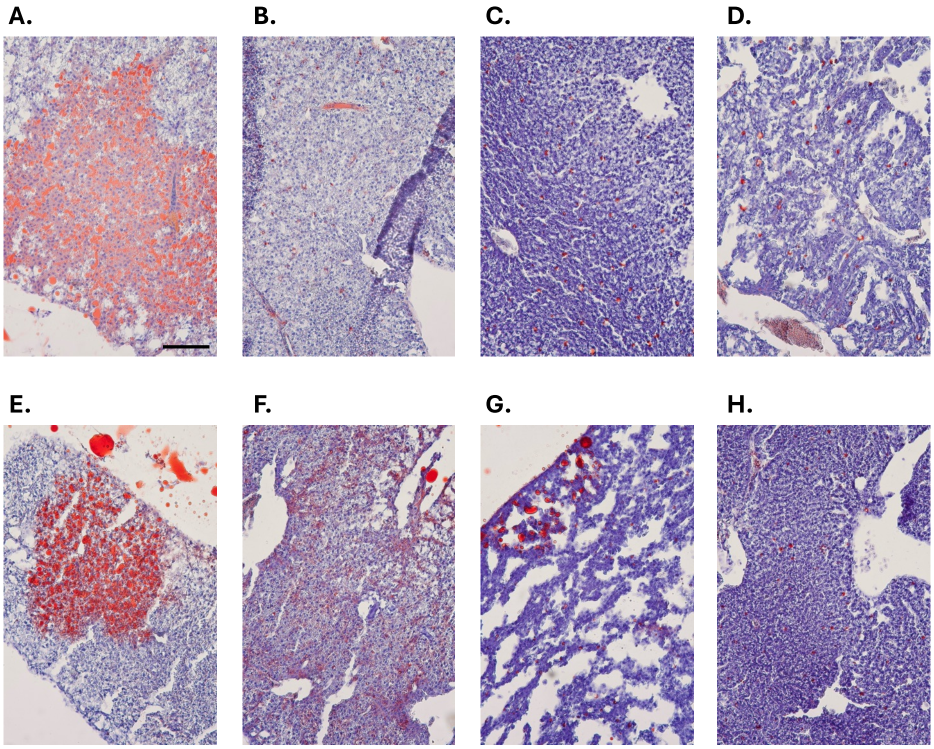

Supplementary Figure 8 Oil-red-O staining on adult liver slides. Fish were fed either 4 % HCD or control diets for 2 weeks and sampled either 2 h after last meal (postprandial fish) or after fasting 3 days (fasted fish). The representative figures were livers from A) postprandial male fed control diet, B) postprandial male fed 4 % HCD, C) postprandial female fed control diet, D) postprandial female fed 4 % HCD, E) fasted male fed control diet, F) fasted male fed 4 % HCD, G) fasted female fed control diet, H) fasted female fed 4 % HCD. Scale = 100 µm.
